# The role of androgens and global and tissue-specific androgen receptor expression on body composition, exercise adaptation, and performance

**DOI:** 10.1186/s13293-025-00707-6

**Published:** 2025-04-23

**Authors:** Sabrina Tzivia Barsky, Douglas Ashley Monks

**Affiliations:** 1 Department of Cell & Systems Biology, Faculty of Arts & Science, University of Toronto, Toronto, ON Canada; 2 Department of Psychology, Faculty of Arts & Science, University of Toronto Mississauga, 3359 Mississauga Road North, Deerfield Hall DH4098, Mississauga, ON L5L 1C6 Canada

**Keywords:** Testosterone, Androgen receptor, Body composition, Skeletal muscle, Adipose, Bone, Exercise

## Abstract

Gonadal testosterone stimulates skeletal muscle anabolism and contributes to sexually differentiated adipose distribution through incompletely understood mechanisms. Observations in humans and animal models have indicated a major role for androgen receptor (AR) in mediating sex differences in body composition throughout the lifespan. Traditional surgical, genetic and pharmacological studies have tested systemic actions of circulating androgens, and more recent transgenic approaches have allowed for tests of AR gene function in specific androgen responsive niches contributing to body composition, including: skeletal muscle and surrounding interstitial cells, white and brown adipose, as well as trabecular and cortical bone. Less well understood is how these functions of gonadal androgens interact with exercise. Here, we summarize the understood mechanisms of action of AR and its interactions with exercise, specifically on outcomes of body composition and muscle function, and the global- and tissue-specific role of AR in regulating skeletal muscle, adipose, and bone morphology. Additionally, we describe the known effects of androgen and AR manipulation on female body composition, muscle morphology, and sport performance, while highlighting a need for greater inclusion of female subjects in human and animal muscle physiology and endocrinology research.

**Supplementary Information:**

The online version contains supplementary material available at 10.1186/s13293-025-00707-6.

## Highlights

- Androgens, produced by gonadal, adrenal, and peripheral tissue conversion in both sexes mediate growth and adaptations of various organ systems partly through the androgen receptor.
- In genetically male mice, global loss of androgen receptor function mutations result in reduced body weight prior to adulthood, late onset obesity beyond into adulthood, and reduced trabecular and cortical bone parameters
- Muscle-specific androgen receptor regulates lean mass, glycolytic but not oxidative skeletal muscle mass and strength, as well as adiposity in both sexes.
- In skeletal muscle, androgen receptor may influence body composition through activation of mitochondrial activity, and through the regulation of genes related to lipolysis, lipogenesis, polyamine biosynthesis, and muscle atrophy.

**Supplementary Information:**

The online version contains supplementary material available at 10.1186/s13293-025-00707-6.

## Background

Sexual dimorphism in body composition, including skeletal size, skeletal muscle mass, adiposity and distribution of adipose tissue are widespread in mammalian species, including humans. These sex differences in body composition are thought to result from a combination of genetic, hormonal and environmental factors. Hormonal contributions are generally attributed to sex differences in levels of circulating gonadal sex steroid hormones, notably including androgens in the form of testosterone. The timing of developmental onset of sex differences in body composition, along with experiments and experiments of nature in which testes are removed or have reduced testosterone secretion have provided ample evidence for testosterone’s actions in organization and maintenance of energy metabolism [1, 2] and muscle and adipose morphology [3]. Further, the widespread use of androgens, androgen mimetics, and selective androgen receptor modulators (SARMs) in clinical and recreational settings to manipulate skeletal muscle size and function, bone strength, and adiposity speaks to the potency of the androgen-AR interaction in regulating body composition.

One interesting facet of these experiments is that experimental gonadectomy (GDX) (and hypogonadism) of males generally indicates anabolic action of androgens on skeletal muscle. However, these effects are skeletal muscle type-specific, with dramatic reduction of the bulbocavernosus and levator ani (BC/LA) perineal muscles, yet more modest atrophy in hindlimb muscles such as soleus, plantaris, gastrocnemius, and extensor digitorum longus (EDL) [4–6]. These observations of variable muscle sensitivity to gonadal androgen, along with observations that AR expression is elevated in the BC/LA relative to hindlimb muscles, has suggested a hypothesis that AR expression at the level of the tissue determines the degree of androgen sensitivity or even dependence of tissues. Indeed, AR expression correlates to androgen sensitivity of a tissue [7], with differences in AR activity shown across various organs in both sexes [8]. In skeletal muscle, baseline AR expression seems to be correlated with training-induced changes of lean body mass (LBM) and fiber cross-sectional area (CSA) in young men [9, 10], with inhibition of AR limiting endurance and resistance training outcomes of hypertrophy in male rats [11, 12].

AR is expressed by myocytes, adipocytes, and osteocytes, and so it is important to define how AR contributes to the development and adaptation of these candidate cells to understand their contribution to lean and fat body composition. Using embryonic genetic knockout strains of AR (ARKO) at the global- and tissue-specific levels, the role of AR in modulating body composition, skeletal muscle morphology, bone characteristics, adiposity, and mitochondrial energy dynamics can be determined. Here, we provide a review of AR, its genomic and non-genomic mode of action, and the role of global- and tissue-specific AR in skeletal muscle, bone, and adipose maintenance in rats and mice throughout the lifespan.

### Androgens and the androgen receptor

#### Androgen receptor overview

The AR protein was first identified and purified in the late 1960’s from rat prostatic tissue [13–16]. The AR gene, located on the X chromosome within loci Xq11-12 [17], was found to be over 90 kb long, and translated into the AR protein at a molecular weight of 110-114 kDa [18]. The AR protein was described to possess three functional domains: a NH_2_-terminal transcriptional regulation domain (NTD), DNA-binding domain (DBD), and C-terminal ligand-binding domain (LBD) [19]. These regions supported the physiological functions of AR as a ligand-dependent transcription factor, allowing testosterone or 5α-dihydrotestosterone (DHT) to bind to AR, stabilize the receptor-DNA complex when dimerized AR binds to DNA at specific androgen-response elements (ARE) [20], and modulate transcriptional activation of androgen-dependent genes [21]. The AR belongs to Subfamily 3 of the Class 1 Nuclear Receptor Superfamily, comprising of steroid receptors which regulate key metabolic processes under a highly conserved DBD, all undergoing homodimerization upon nuclear translocation (Class I: progesterone receptor (PR), glucocorticoid receptor (GR), mineralocorticoid receptor (MR), and Class II: estrogen receptor (ER) alpha and beta) [22, 23]. Mutations in the AR gene have been implicated in conditions which affect body composition, including androgen insensitivity syndrome (AIS) [24, 25] and Spinal and Bulbar Muscular Atrophy (SBMA) (ie., Kennedy’s Disease) [26–30]. AIS is an umbrella term for mutations which result in partial or complete loss of AR function in XY individuals. SBMA on the other hand is caused by expansion of polyglutamine (CAG) repeats in the NTD and is associated with both loss and gain of AR function and results in progressive degradation of motor neurons, causing muscle weakness [31, 32]. In both AIS and SBMA, changes in body composition and muscle strength consistent with loss of AR function are observed [33–35]. Although AR-androgen action was most known for its crucial role in the development of the male reproductive system and sexual maturation, the ubiquitous expression of AR in nearly all major organ systems [36, 37] highlighted the importance of this steroid receptor in the growth and adaptation of many tissues, including muscle, bone, and adipose.

### Androgen synthesis, secretion, and peripheral metabolism

Activation of AR is dependent of ligand-binding of androgens, which are produced mainly by the gonads and adrenal glands, through the highly regulated hypothalamic pituitary gonadal or adrenal axis. The neuromodulator of pubertal onset, kisspeptin, acts on its receptor (KiSS1R) located on the gonadotropin-releasing hormone (GnRH) neuron in the hypothalamus, promoting the pulsatile secretion of GnRH into portal circulation [38]. Within the anterior pituitary gland, bound GnRH results in the release of follicle-stimulating hormone (FSH) and luteinizing hormone (LH), promoting processes of sexual maturation at the level of the testes and ovaries [39–41]. Within the testes, increased concentration of LH causes secretion of androgens from the Leydig cells, and increased androgens and FSH initiates spermatogenesis in the Sertoli cells [42]. In response to elevated LH, androgens are produced in the Theca cells of the ovary, where they act as precursors for estrogen production, undergoing aromatization into the main estrogen, 17β estradiol (E2), within Granulosa cells. FSH elevations promote androgen aromatization to estrogens in the Granulosa cells, a process which contributes mainly to folliculogenesis and oocyte maturation [43]. In both males and females, androgen secretion through gonadal or peripheral production is maintained through negative feedback, wherein excessive testosterone (or primarily excessive estrogens in females) in circulation suppresses LH production at the level of the pituitary gland and GnRH from the hypothalamus.

Testosterone is the major circulating androgen in males, with its production predominantly occurring in the Leydig cells of the testes via precursors, including cholesterol and androstenedione [43, 44]. The adrenal glands play a significant role in androgen production (alongside the ovaries) in women, where dehydroepiandrosterone (DHEA), as well as small amounts of cortisol and E2, are secreted from the *zona reticularis* of the adrenal cortex [45, 46]. Rodents, however, produce little to no adrenal androgens [47]. Peripheral conversion of DHEA to testosterone in the adrenal glands occurs via 3β- and 17β-hydroxysteroid dehydrogenase (3β-HSD, 17β-HSD), and is mostly seen in females. Peripheral conversion of DHEA to testosterone can be seen in the brain, liver, breast, blood cells, skin, adipose, adrenal glands, gonads, and accessory sex organs. Additionally, testosterone can be converted to its highly androgenic metabolite, DHT, and to E2 in the adipose, ovaries, and brain, through 5α-reductase and aromatase, respectively [48–50].

In human males, total testosterone ranges from 0.069–5.73 nmol/L at 8–11 years of age, increasing to ranges of 0.104–28.66 nmol/L at 12- to 17-years of age [51]. In young adult males, the average testosterone levels range from 7–35 nmol/L [52], with most studies pointing to a steady 1–2% decline of bioavailable testosterone per year beyond 30 years of age [53–55], while a few studies report a minimal decline in circulating testosterone between 35- and 65-years of age [56–58]. There is a steady decrease in both total and free testosterone from the third to ninth decade of life in healthy men, with slightly steeper declines in free testosterone [59, 60], which correspond with the trend of increased SHBG throughout the male and female lifespan [54, 61]. Female values are approximately 15-fold lower at all points across the lifespan, with prepubertal, pubertal, and adulthood total testosterone levels ranging from 0.035–2.01 nmol/L [51]. Female testosterone levels seem to reach their peak between 20- and 25-years of age, declining steadily with age [62].

Bioavailability of free testosterone in the blood was shown to be minimal at only 2–3% in males, as the remainder of gonadal testosterone is sequestered strongly by high affinity steroid hormone binding globulin (SHBG) (~ 44% in males and ~ 66% in females), with a smaller amount (~ 50% in males and ~ 30% in females) weakly bound to low affinity albumin or corticosteroid binding globulin [63]. In contrast, the majority of adrenal androgens circulate bound to albumin (~ 90% in both males and females), while a small percentage are bound to SHBG (~ 3% in males and ~ 8% in females) [63]. Free testosterone is lipid soluble and upon crossing through the plasma membrane, heat shock protein (Hsp70/90) inhibition of AR is removed, and testosterone or DHT are free to bind to AR [64, 65]. Androgen activation can induce two canonical modes of action, including rapid, non-genomic mechanisms which modulate secondary signaling pathways in an AR-dependent or -independent manner, or through genomic, AR-dependent mechanisms via nuclear translocation and transcriptional regulation of AR-dependent genes.

### Tissue specificity of AR

The prostate, epididymis, seminal vesicle, and testes were shown to be highly androgen-responsive tissues considering the key role of testosterone and AR in regulating male sex differentiation and external/internal genital development [66–68]. However, skeletal muscle as an androgen target was first hypothesized in 1889 after published descriptions of increased forearm flexor strength were followed by subcutaneous injection of rodent testicular aqueous solutions [69]. Brown-Sequard’s self-reported claims were summarized to be that of a placebo effect [70] as his daily injections were measured to have approximately 32,000-fold less testosterone than the average daily secretion of a healthy male at 186 ng/day compared to 6 mg/day, respectively [71]. Nonetheless, his seminal work was thought to pioneer the emergence of endocrinology as a scientific discipline and promoted the clinical interest in pharmaceutical use of sex steroids in human health and disease.

Various animal models were used to investigate muscle targets of androgen action, including the temporal muscle of the guinea pig [72], the dilator laryngis muscle of *Xenopus laevis* [73], the syringeal muscles of song birds [74], as well as BC/LA of rodents [75, 76]. To our knowledge, the first mention of the “androgen receptor” in relation to sex-specific changes in tissue morphology was made by Neumann and colleagues in 1966, where treatment of pregnant rats with cyproterone acetate (a potent anti-androgen) resulted in genetic male pups with incomplete scrotal development and reduced perineal width, indistinguishable from genetically female pups [13]. Mainwaring [14, 15] reported the presence of AR in rat prostate, then described as a protein which bound DHT more readily than testosterone or other anabolic hormones. Later, the group described the purification of the AR protein from rat tissue [16]. In 1972, Jung and Baulieu identified a similar “testosterone-binding cytosol receptor” in rat LA muscle and prostate, citing that compared to prostate, muscle had overall lower receptor counts but greater binding affinity to testosterone than DHT [76]. This tissue-dependent binding affinity of androgens and AR protein was supported by other groups which identified 60-times greater DHT binding sites in prostate than skeletal muscle [77–79]. Loss of endogenous circulating testosterone via castration has long shown the atrophic outcomes of muscle [72] and marked increases in adiposity [80, 81], which can both be reversed by testosterone treatment, even in the presence of pharmacological 5α-reductase inhibition [82]. Androgen sensitivity varies even across different skeletal muscles, with higher testosterone responsiveness in shoulder versus other upper-limb muscles of *Xenopus laevis* [83], in trapezius versus vastus lateralis of healthy, untrained males [84], and in LA versus EDL of young male rodents [7, 85]. At basal levels, AR protein is highly expressed in myonuclei and satellite cells (SCs), as well as fibroblasts and in mast cells proximal to capillaries in the connective tissue of vastus lateralis sections from young 18- to 35-year-old males [86]. Quantification of AR-positivity was significantly higher in myonuclei and fibroblasts of post-natal day (PND) 60–90 male LA compared to EDL [7, 85]. Similarly, AR gene expression was approximately 400% higher in LA than in gastrocnemius, soleus, or EDL in 12-week-old male mice [87]. Although AR gene and protein expression in male mouse gastrocnemius is dynamic and has been shown to increase from E18.5 to 3-months of age [87] and decrease from 3- to 18-months of age [88], lifespan data observing changes in AR expression across multiple muscles in males and females is limited. We have recently showed that AR protein expression varies between EDL and soleus of males and females during early life (ie., PND1 and PND10), sexual development (ie., PND21, PND42, and PND70), and adulthood (ie., 6-months, 8-months, and 12-months), highlighting the dynamic nature of AR by age, sex, and muscle fiber type [89].

In 1937, Moore and Dorothy [90] showed that androgen treatment rescued castration-induced atrophy of prostate and seminal vesicles in pre- and post-pubertal male rats. In males, production of androgens in the gonads and locally at the prostate plays a role in prostate development, however excessive activation of AR via DHT binding can lead to aberrant cell cycling, pathological prostate growth, and the progression of benign prostatic hyperplasia (BPH) or prostate cancer [91]. Androgen deprivation therapy via 5α-reductase inhibitors can suppress the pathophysiological androgenic response. However, changes to AR activation via AR point mutations, AR overexpression, altered androgen biosynthesis, AR variants, or altered AR transcriptional machinery support the progression of castration-resistant prostate cancers (CRPC), and limit the efficacy of androgen deprivation therapies [92–95]. The clinically significant nature of prostate cancer has led to the discovery and development of several anti-androgen and AR-targeted therapies, altogether deepening the knowledge of AR-mediated action in prostate cells and its involvement in cell cycling and mitosis [96, 97]. Although adult skeletal muscle is debatably post-mitotic [98], AR and androgenic regulation of cell cycle activity/exit in the prostate has opened discussions for comparing underlying mechanisms of AR-mediated determination and adaptation of AR-expressing SCs within the myofiber [86, 99, 100].

### Genomic mode of action

**Fig. 1:**
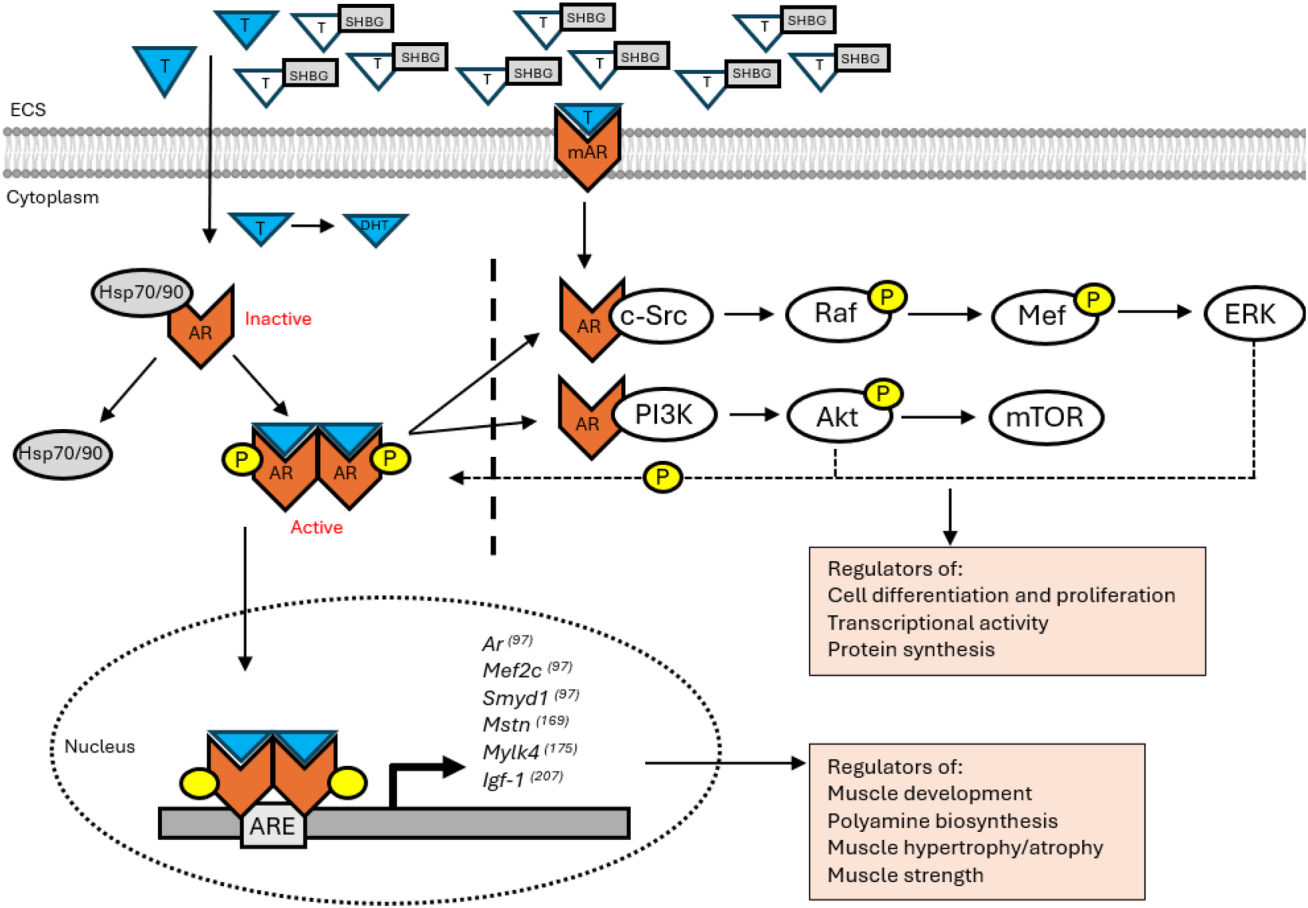
Androgen and androgen receptor (AR) genomic and non-genomic action, and identified genes in skeletal muscle with direct regulation by AR binding to ARE consensus sequences. *ECS* extracellular space, *T* testosterone, *SHBG* steroid hormone binding globulin, *mAR* membrane-bound AR, *DHT* 5α-dihydrotestosterone, *Hsp70/90* heat shock proteins 70/90, *P* phosphorylation, *c-Src* proto-oncogene tyrosine protein kinase, *Mef* myocyte enhancer factor, *ERK* extracellular signal-regulated kinase, *PI3K* phosphatidylinositol-3 kinase, *Akt* protein kinase B, *mTOR* mechanistic target of rapamycin, *ARE* androgen response element, *Smyd1* histone-lysine N-methyltransferase, *Mstn* myostatin, *Mylk4* myosin light chain kinase 4, *Igf-1* insulin-like growth factor-1

Steroid hormone receptors are characterized by their capacity to translocate into the nuclear envelope, bind to targeted DNA sequences, and promote transcriptional activity to mediate tissue development, growth, or metabolism. In the cytoplasm, unbound AR maintains sequestration by heat-shock chaperone proteins, notably Hsp70, Hsp90, and Hsp40 [65, 101]. While the expression of some Hsps was greater in male versus female rat quadriceps, there was no sex difference in Hsp70 content at baseline [102]. Free, unbound circulating androgens permeate the cell membrane binding to AR at the LBD, which is situated within the C-terminal domain of the AR protein (Fig. 1). This results in conformational change of AR, dislodging Hsps and exposing the nuclear localization signal (NLS), located at the junction of the DBD and hinge region [103], and coordinating nuclear pore transport via importin-α binding [104]. Nuclear import, guided by the NLS, follows the organization of a dimerized AR complex which uses the two zinc-finger motifs of the DBD to bind the AR homodimer complex selectively to ARE half-sites [105]. Taken together, this sequence of events allows AR to act as a transcription factor, alongside other coactivators and polymerases, to promote or repress gene transcription of androgen-dependent genes.

Advanced techniques such as chromatin immunoprecipitation sequencing (ChIP-Seq) have been used to identify genome-wide AR-binding on AREs in androgen-insensitive prostate cancer cell lines [106] and in the mouse epididymis [107]. Wilson and colleagues [108] identified amplified genes implicated in steroid biosynthesis and lipid metabolism, including but not limited to 17β-HSD and lipoprotein lipase (LPL) in androgen-insensitive prostate cancer cell lines. Additionally, the identified AR-target genes showed enrichment in pathways involved in cell cycle, DNA recombination and repair, epigenetic regulation, and amino acid metabolism, with specific enrichment of mechanistic target of rapamycin complex 1 (MTORC1) and mitogen activated protein kinase (MAPK) signaling [108]. Coordination of dimerized AR to AREs was shown to utilize additional transcription factors, including Krüppel-like factors (KLF), forkhead box K1 (FOXK1), and sterol regulatory element binding factor (SREBF) [108], which are respectively involved in mediating gene expression related to muscle atrophy [109], cell proliferation [110], as well as lipogenesis [111].

There are sex differences in genomic AR binding within cultured mesenchymal cells from male prostate and female urethra of rats, wherein female tissue presented enrichment of AR at proximal promoter regions and male enrichment was at intergenic regions [112]. Furthermore, genomic AR action seems to influence sexual dimorphism of the urogenital tract via Wnt/β-catenin pathways, as ChIP-Seq showcased AR-binding proximal to transcription start sites on estrogen receptor alpha (*ESR1*) and R-spondin 2 (*RSPO2*) genes [112]. Although there is a greater focus on androgen-dependent genomic regulation of transcriptional events in reproductive and androgenic tissues, there are few works which identify AR-binding sites in muscle tissue. In a genome-wide ChIP analysis of primary myoblasts, Wyce and colleagues [113] identified DHT-dependent AR binding at myocyte enhancer factor 2c (*MEF2c*) and *SMYD1* genes, which play significant roles in fiber type regulation and sarcomere integrity (Fig. 1). Expression of skeletal alpha actin (α-actin) indicated terminal differentiation of the myofiber and had shown androgen- and AR-specific upregulation [114, 115]. Although there is no identified ARE on the α-actin promoter, AR cooperatively bound to serum response factor (SRF) and its response element to synergistically co-activate the α-actin gene in C2C12 myoblasts under exogenous androgen treatment [116].

### Non-genomic mode of action

In addition to nuclear translocation, androgen-bound AR can activate several secondary messenger pathways implicated in cellular growth. Time-to-effect is longer for classical genomic signaling, as it involves recruitment of cell machinery to initiate gene transcription and coordinate translation of mRNA into proteins. However, androgens can induce rapid, non-genomic effects characterized by their time of on-set (ie., seconds-minutes) and speed of downstream effects depending on AR-dependent or -independent activation. Briefly, administration of testosterone and synthetic androgens in cell cultures provided strong time-course evidence of androgen action on intracellular calcium (Ca^2+^) homeostasis, as well as activation of protein kinases A/C (PKA/PKC), MAPK, phosphatidylinositol-3 kinase (PI3K), protein kinase B (Akt), and cAMP response element-binding protein (CREB) [117].

In a bell-curved dose-dependent manner, testosterone administration increased intracellular calcium influx within 5-s, and inositol phosphates (IP), monoacylglycerol (MAG), phosphatidic acid (PA), and diacylglycerol (DAG) formation within 10–20 s in osteoblasts isolated from parietal bones of PND2 male rats [118]. Time-course studies in myotube cultures of neonatal rat hindlimbs showed that androgens, but not E2 or progesterone, increased calcium transience and inositol triphosphate (IP3) production in under 1-min, with dose-dependent increases of extracellular signal-regulated kinase 1/2 (ERK1/2) protein phosphorylation within 5-min through Ras and MEK activity [119]. These androgen-mediated effects remained after AR antagonist administration in myotube cultures, highlighting AR-independent action of androgens and the likely utilization of an androgen-responsive G-couple protein receptor in fast, non-genomic signaling [119, 120]. Androgens and AR seem to promote CREB activity in muscle, as CREB acted as a co-activator of AR-dependent transactivation [121] and androgen treatment increased CREB activity in cultured myotubes via AR action [119]. DHT treatment in mouse osteoblasts increased Akt activity within 15-min and induced AR-dependent maximal nuclear translocation of Akt kinase within 40-min of treatment [122]. Additionally, androgen treatment stimulated Akt and PI3K activation via AR- and proto-oncogene tyrosine protein kinase (Src)-dependent mechanisms within prostate and breast cancer cell lines [123–126]. Sex hormone and androgen-metabolite induction of Src, Raf-1, ERK1/2, MEK, and CREB activation [126, 127] were mediated by androgen and estradiol treatment-induced AR association (within 1-min) to the SH3 kinase domain of Src in cancer cell lines [124]. Androgen time-to-effect on activation of cell growth mediators varies across different tissue types, as well as healthy or cancer cell lines used. Vascular smooth muscle cells cultured from 12- to 16-week-old rats exhibited an increase in phosphorylated Src within 120-min [128]. Taken together, non-genomic action of androgens and AR are likely regulated through activity of Src, Ras/Raf-1, PI3K/Akt, MEK, ERK1/2, and CREB (Fig. 1).

Androgens can activate distinct pathways implicated in muscle protein synthesis (MPS) and lipolysis. Although androgen-mediated increases in regulators of MPS and decreases in regulators of adipogenesis cannot directly indicate changes in muscle or adipose mass, the time-course data does provide a basis for understanding how androgen-AR action can affect body composition with repeated exposure, activation, or signaling. The role of androgens in promoting muscle protein synthesis was first investigated in 1965, where Breuer and Florini [129] revealed that 7-days of testosterone treatment rescued the castration-induced 50% reduction of skeletal muscle ribosome activity, measured by incorporation of a radioactive [^3^H]leucine into protein. This androgen-mediated effect on elevating protein synthesis rates was seen as well in healthy males following a single intramuscular testosterone injection [130]. mTORC1 and its downstream targets, 40S ribosomal protein S6 kinase 1 (S6K1) and eukaryotic initiation factor 4E-binding protein 1 (4EBP1), were required to stimulate skeletal muscle hypertrophy in rat hindlimb muscles [131] and promote load-induced muscle mass gain [132, 133]. Altamirano et al. [134] showed that 48-h of testosterone treatment in cultured neonatal rat cardiomyocytes increased phosphorylation of mTORC1, S6K1, 4EBP1, ERK1/2, and Akt, and that mTORC1 activity was required for androgen-induced muscle hypertrophy. In skeletal muscle, 7-days of androgen treatment following castration in mice increased AR expression, and protein phosphorylation of mTOR, p70S6K, 4EBP1, and Akt above sham groups [135]. Moreover, pharmacological induction of hypogonadism in young males decreased MPS rates and phosphorylation of mTORC1, S6K1, 4EBP1, and Akt after 6-weeks of resistance exercise, likely contributing to the phenomenon of anabolic resistance seen in aged men [136].

Originally studied in relation to castration-resistant prostate cancer progression, oncogene β-catenin and its activation of the Wnt pathway was shown to bind AR and enhance its transcriptional activity in prostate cancer cell lines [137, 138]. However, an interest in testosterone-induced reduction of fat mass in men [139] led to the investigation of androgen-AR action in adipose, which revealed higher AR expression and androgen binding sites in preadipocytes compared to mature adipocytes in a depot-specific manner [140, 141]. Singh and colleagues [142] showed that treatment of mouse preadipocytes with androgens resulted in a dose-dependent reduction of adipogenic transcription factors, CCAAT-enhancer-binding proteins (C/EBP-δ, C/EBP-α) and peroxisome proliferator-activated receptor gamma (PPAR-γ2), in addition to increased AR-dependent β-catenin nuclear translocation. In multipotent cells, androgen treatment promoted β-catenin nuclear translocation within 30-min, which was shown to coordinate the testosterone-induced upregulation of myoblast determination protein 1 (MyoD) and myosin heavy chain 2 (MHC2) [143]. Altogether, androgen-AR action in tissue anabolism and catabolism may be transduced through the non-genomic activation of mTORC1 and β-catenin/Wnt pathways, respectively, and their downstream activators of transcriptional activity.

Although circulating testosterone is largely thought to affect body composition mainly through androgen-AR, estrogenic actions resulting from local aromatization to E2 also affects body composition. Ovarian estrogens are well-known to contribute to sex differences in testosterone and estradiol, mediating sex-specific deposition of subcutaneous and visceral fat mass, with E2 levels being protective of visceral adipose expansion and inflammation [144]. Testosterone has a seemingly inverse relationship with fat mass, wherein obese men have markedly lower levels of circulating testosterone [145]; weight loss is proportional to testosterone changes in men [146]; and obesity is a major contributor to age-related declines in testosterone [147]. Although the relationship between fat and testosterone levels may be compounded by comorbidities, some research suggests that one of the underlying mechanisms governing this is the coordinated action of E2 activation of estrogen receptor alpha (ERα) through increased production of E2 at the level of adipose tissue via testosterone aromatization [148]. Adipocytes, which undergo hypertrophy, hyperplasia, and chronic macrophage infiltration during the progression of obesity express more CYP19 transcripts, leading to higher levels of aromatase [149], likely contributing to the greater expression of E2 from conversion of testosterone. The CYP19A1 gene encodes aromatase, with its expression being highest in the testis, the hypothalamus, and the extrahypothalamic regions [148]. In obese men, higher circulating levels of estradiol will in turn negatively regulate the hypothalamic pituitary axis, lowering FSH and LH, and subsequent testosterone production from the gonads [150, 151]. However, E2 and ERα seem to be at least in part required for normal adipose development and function in males [152]. E2 supplementation in high-fat diet fed male mice results in anti-obesogenic effects in adipose tissue [153]. Aromatase knockout (ArKO) inhibits endogenous E2 production and results in an obese phenotype in both male and female mice [154]. Global knockout of ERα in both female and male mice resulted in increased white adipose tissue (WAT) mass (ie., epididymal, perirenal, and inguinal) but not brown adipose tissue (BAT) mass measured over the first year of life [152]. Moreover, ERαKO resulted in significantly increased adipocyte size and count in epididymal and perirenal adipose depots at PND180, altogether highlighting the importance of E2 and ERα in maintaining sex-specific adipose development. Testosterone’s capacity to aromatize to E2 and activate ERα may serve as the mechanism for testosterone-mediated fat loss. Kim and colleagues [148] found that testosterone but not DHT improved body mass, fat mass, lean mass, and WAT mass outcomes following chemical castration from 16- to 36--weeks of age in WT male mice. However, an inducible knockout of ERα from extrahypothalamic regions inhibited those testosterone-mediated changes in total and fat body composition, but not lean mass, suggesting that conversion to E2 is required for testosterone-induced fat catabolism.

Along with gonadal hormones, sex chromosomes have also been identified as major factors mediating sex differences in body composition. However, understanding the influence of genetic sex on phenotypic and metabolic outcomes in lean and non-lean tissue requires a decoupling of biological sex from gonadal hormone profile. Several studies have used the transgenic mouse Four Core Genotypes model (FCG) to disentangle the different contributions by either gonads or sex chromosomes in established sexually dimorphic phenotypes [155, 156]. The FCG model utilizes the deletion of SRY from the Y chromosome in XY mice and the overexpression of SRY in XX mice, resulting in progeny that are XX with ovaries, XX with testes, XY with testes, and XY with ovaries. This allows for comparison of a trait’s influence by either the gonads, the chromosomal complement, or their interaction. Ramirez and colleagues [157] observed that chromosomal sex affects lean mass at 4-months of age and fat mass between 2- and 4-months of age. Specifically, XY mice with ovaries have higher lean body mass percent (LBM%) and lower fat body mass percent (FBM%) than XX mice with ovaries or testes at 4-months. For muscle mass maintenance, the group confirmed that gonadal sex plays a stronger role such that all mice with testes had greater absolute mass of EDL, gastrocnemius, TA, and quadriceps. However, prior to the full maturation of the gonads and their hormonal secretions during puberty, there are sex differences in gene transcription in mouse embryonic stem cells [158] as well as sex differences in fetal body size and proportion [159], revealing that genetic sex and chromosomal expression can influence both tissue morphology and gene expression. Although the influence of sex chromosomes on skeletal muscle mass and fat deposition seems to be minor and age-dependent, it is relatively understudied, with the dominant driver of sex differences in these tissues and the brain pointing to gonadal hormone production [157, 160, 161].

### Androgen receptor manipulation in research

#### Transgenic mutants: global androgen receptor knockout

Clinical observations of androgen insensitivity syndrome were first presented by Morris in 1953 [162] (then described as testicular feminization) in humans, and later in rats [163] and mice [33]. Cases of androgen insensitivity can be classified as complete, partial, or mild, and diagnoses are made by observation of female external genitalia in a XY karyotype male fetus (ie., complete androgen insensitivity), gynecomastia and atypical genitalia at puberty (ie., partial androgen insensitivity), or unaffected genitalia yet presence of male infertility (ie., mild androgen insensitivity). Complete androgen insensitivity was discovered and propagated in a substrain of rats, lending an in vivo model to study the molecular underpinnings of androgen insensitivity and the influence of lost AR function on sex development, aptly named the testicular feminized (*Tfm*) rat. *Tfm* males develop testes, which remain undescended in the inguinal canal, appearing to have immature Sertoli cells limiting the progression of spermatogenesis [164] and hyperplasia of Leydig cells allowing for normal to excess androgen production [165]. Additionally, *Tfm* males do not develop male accessory sex organs (ie., prostate, epididymis, ductus deferens, seminal vesicles). Studies attempting to identify mechanisms of androgen insensitivity in the *Tfm* rat revealed that cytoplasmic AR was decreased in target organs [166, 167] and had reduced binding capacity for androgens [168], which resulted from a single base mutation in the AR gene [169]. The limitations in the *Tfm* model included male sterility and no opportunity to study females with complete aberrant AR function, as female carriers of *Tfm* are genetic mosaics for androgen insensitivity [170]. This brought forth the production of the androgen receptor knockout mouse (ARKO) [68].

In 1981, Sternberg and Hamilton [171] characterized a site-specific DNA recombination system, identifying a locus of crossing over (x) in P1 phage (loxP) and a recombinase gene (Cre) as two required components of DNA recombination. Later, the Cre-loxP system was shown to be effective in mammalian cell lines [172], leading to further development into a mouse germ line to reveal successful transmission of gene deletions across offspring [173]. Considering the limitations of the *Tfm* model, Yeh et al. [68] created the first male and female ARKO mice by utilizing the Cre-loxP system to flank exon 2 of AR with two loxP sites, catalyzing the excision of the flanked sequence only where Cre was expressed. To drive Cre-recombinase expression in all cells, floxed AR mice were mated with Cre mice carrying Cre expression under a β-actin (ACTB) promoter, producing offspring with non-functional AR protein due to a frame-shift mutation within the AR DNA-binding domain. Since then, several founding lines of ARKO mice have been established and used by other groups, with Cre expression driven by distinct promoters, including human cytomegalovirus (CMV) [87, 174–184], phosphoglycerate kinase 1 (PGK) [185–192], and ACTB [68, 193–197] (See Supplementary Table 1.0). Additionally, the flox sites have differed across these transgenic lines [198], with some groups inserting loxP sites at exon 1 resulting in a frame-shift mutation [174], exon 2 resulting in a frame-shift mutation [68, 185], and exon 3 resulting in an in-frame deletion [177]. To note, exon 1 encodes the NTD, whereas exons 2 and 3 encode the DBD [199]. Compared to the frame-shift mutations which completely nullified AR protein translation, the in-frame deletion of exon 3 disrupted only the zinc-finger of the AR DBD but allowed for the maintenance of non-DNA binding-dependent AR action [177, 200]. The varying strategies of developing an ARKO line are important to note as characteristics such as the presence of the selection cassette, the promoter selected for transgene integration, the position of the loxP sites, or the utilized mouse background all likely contribute to differences in tissue selectivity, AR activity, and the resulting phenotypes [178, 201].

A significant collection of studies revealed the effects of global and cell-specific AR ablation in the testes, ovaries, and male accessory sex organs on promoting and maintaining primary sex characteristics, sexual behavior, and androgen-mediated maturation of the gonads [202]. While previous work had sufficiently shown that activation of AR through endogenous and exogenous ligands sufficiently increased lean mass with some adipose sparing capacity alone or in the presence of exercise stimulus, the function of AR in regulating body composition, voluntary exercise, or forced exercise outcomes was less clear. In the first global ACTB-driven ARKO mouse line, Yeh and colleagues [68] revealed that functional AR expression regulates levels of circulating testosterone, masculinization of external genitalia, normal bone phenotype, and masculinized body weight. Other works from the same group later identified that functional AR was protective against obese phenotypes, where ARKO male mice had increased body mass, skeletal muscle triglycerides, relative WAT mass, and WAT CSA in adulthood [194], as well as increased WAT and BAT adipocyte size at 12-weeks of age [197]. Interestingly, ARKO in the PGK-driven mouse line did not influence fat body mass (FBM) or adiposity in adulthood, but rather presented a modest reduction in LBM and several bone parameter measures [186, 188, 189]. These studies, and others from the same group, presented consistent reductions across total body mass (TBM) in their PGK-driven ARKO mice [185, 187], similar to that of ACTB-driven ARKO mice [68, 194]. Likewise, ARKO in the CMV-driven mouse line had shown reduced TBM in males across adulthood [177–181, 184], with some of those works also highlighting a modest reduction in hindlimb muscle mass and increased subcutaneous and visceral fat depot mass [178, 180, 184]. Though the outcome variables differed between these studies with some combination of total, muscle, adipose, and/or bone parameters measured, a relatively consistent pattern was seen across ARKO males, such that functional AR was necessary to maintain normal body mass, adiposity, trabecular and cortical bone composition, and to a more modest degree, lean mass and/or hindlimb skeletal muscle mass in adulthood. Ultimately, this transgenic model opened avenues to study the tissue-specific roles of AR by using the Cre-loxP system with cell-specific gene promoters (See Supplementary Table 1.0).

### Transgenic mutants: cell-specific androgen receptor knockout

Through global ARKO models, it became clear that androgen-AR activity contributed to notable modulation of skeletal muscle, adipose, or bone phenotype, which drove further exploration into the specific roles of AR within those cells. Body composition outcomes were studied by a handful of mouse models utilizing cell-specific promoters driving ARKO within SCs [191, 192, 203], myoblasts [183, 204], myocytes [187, 205–208], myofibers [183, 204, 209], neurons [192, 210, 211], osteoblasts [212–214], osteoclasts [182], and adipose [215, 216]. This subsection will focus on the changes to body composition in response to tissue-specific ARKO.

#### Bone-specific ARKO

Body composition could be defined by several compartment models, the first being the two-compartments of FBM and LBM introduced by Behnke and colleagues [217–219], the three-compartments of FBM, LBM, and mineral content derived by Siri and colleagues [220, 221], and the four-compartments of FBM, total body water, metabolic tissue, and mineral content [222, 223]. Thus, bone parameters, including length, density, thickness, and volume, contribute to the overall picture of body composition. Furthermore, due to the highly plastic nature of the skeletal system in response to hormonal flux during physiological stressors of puberty, aging, and menopause, as well as supraphysiological stressors of androgen or SARM doping, osteocytes are an important target for possible adaptation via androgen-AR action.

The osteogenic-ARKO and SARM literature studying skeletal morphology has classically used the distal femur and lumbar vertebrae because of their load-bearing potential with high trabecular bone content properties [224], which make these tissues dynamic and highly involved in tissue remodeling via bone resorption and formation. The seminal work of Albright and Recfenstein in 1947 first highlighted the relationship between circulating androgens and bone maintenance [225], opening the doors to later works which supported the notion that androgens were necessary for puberty-mediated bone growth [226] and were associated in governing sex differences in bone characteristics [227]. Although SARM literature had focused on the benefit of AR activity in bone composition and overall anabolism in aging and disease, there was little understanding of the specific role of bone-AR in bone remodeling. In 2007, Notini et al. [212] utilized rat type 1a1 collagen (Col1a1) promoter to create the first mature osteoblast-specific ARKO, identifying some minor effects on femur and vertebral body morphology at 6-, 12-, and 32-week-old male mice. The group revealed that reliance on functional AR within osteoblasts was bone-dependent, visualized by greater and earlier detriment to skeletal morphology in vertebrae but not the femur. Osteoblast-ARKO caused decreased vertebral body bone trabecular number and connective density, and increased trabecular separation at 12- and 32- weeks. In contrast, the femur was less affected, with no changes to femur length, trabecular thickness, or trabecular number at any measured timepoint, yet at 32-weeks, males exhibited decreased bone volume and increased trabecular separation. Using an osteocalcin-Cre driver to ablate AR from mineralizing osteoblasts, the same group [213] later identified that osteoblast-ARKO did not change bone mineral density (BMD) or trabecular bone characteristics in early ossifying bone of the distal femur in male mice at 6-, 12-, or 24-weeks of age, yet did reduce bone volume and trabecular number in mature bone at 6- and 24-weeks. Lumbar vertebra from osteoblast-ARKO male mice showed modest decreases in trabecular bone number and increases in trabecular bone thickness at all growth and adult ages, independent of changes in body mass [213], in contrast to the greater deficit seen across vertebrae versus the femur in the group’s previous osteoblast-ARKO work [212]. Trabecular bone seemed to be the major target of AR action, as another osteoblast-ARKO driven by osterix-Cre showed modest worsening of trabecular bone in lumbar vertebrae but neither trabecular nor cortical bone of the femur in 10-14-week-old male mice, compared to controls [214]. In this work, the authors reported no change in dual energy X-ray absorptiometry (DXA)-measured BMD or bone mineral content (BMC), although this method is incapable of differentiating between trabecular and cortical bone. AR signaling specifically in osteoclasts was observed to be less important in maintaining either trabecular or cortical bone in cathepsin K-Cre driven ARKO mice at 12- and 32-weeks of age [182]. Overall, it seems that AR has a stronger role in maximizing bone morphology within osteoblasts versus osteoclasts, and this regulation appears to be dependent on the observed bone, trabecular parameters, and bone maturity.

#### Adipose-specific ARKO

Surprisingly, the pool of adipose-specific ARKO literature is smaller than that of bone- and skeletal muscle-specific ARKO, even considering the well-established contribution of circulating androgens in sex differences in adiposity and body fat distribution [228]. After identifying that global ARKO resulted in the decrease of subcutaneous adipose depot mass in 8-week-old male mice [68] and late onset obesity in male mice beyond 12-weeks of age [194], the same group developed an ARKO mouse line with an adipose-specific knockout using an adipocyte Protein 2 (aP2)-Cre driver to identify the contribution of adipose-specific AR in fat maintenance [215]. In 20-week-old male mice, Yu et al. [215] found that AR deficiency specifically in adipose did not perturb body weight, or epididymal or perirenal WAT adipocyte area, triglyceride content, or adiposity index. However, adipose-ARKO resulted in increased mRNA expression of peroxisome proliferator-activated receptor gamma coactivator 1-alpha (PGC1α), uncoupling protein 2 (UCP2), and fatty acid mitochondrial transporter (CPT1) in epididymal WAT, suggesting that adipose-specific AR may be involved in lipid mobilization and fatty acid metabolism. Similarly, McInnes and colleagues [216] observed no effect of aP2-Cre mediated adipose-ARKO on body mass at 6- and 12-months of age, as well as no effect in WAT depot mass or adipocyte CSA at 3- and 12-months. Yet, mRNA expression of prominent lipolysis genes, adipose triglyceride lipase (ATGL), fatty acid synthase (FAS), hormone sensitive lipase (HSL), and LPL, were significantly upregulated in WAT of adipose-ARKO males at 3-months [216]. Overall, adipose-AR seems to regulate lipid metabolism, with lesser impacts on adipose phenotype during adulthood.

#### Skeletal muscle-specific ARKO

Skeletal muscle is the largest multinucleated tissue in the mammalian body and possesses a hierarchical structure consisting of the muscle fiber, muscle fascicles, myofibers, and myofibrils [229]. Each myofibril contains repeating contractile units of the muscle, the sarcomere, made up of overlaying units of actin and myosin located between Z-disc scaffolds [230], which can be observed using Transmission Electron Microscopy (TEM) [231, 232]. Using standard light microscopy, the muscle fascicle can be visualized to show a cross-sectional slice of each muscle fiber, their surrounding connective tissue, and the embedded myonuclei. During embryonic development, skeletal muscle is formed following progenitor cell commitment to myogenic lineage, founding the myoblast and subsequent development of the myocyte, and the details of this process in relation to androgens and AR are reviewed elsewhere [99]. Herein, this subsection describes the outcomes of AR ablation at various levels of skeletal muscle organization on body composition.

The role of myocyte-specific AR in mediating both muscle and fat phenotype was first described in 2009 by Ophoff and Van Proeyen et al. [187] where it was shown that muscle creatine kinase (MCK)-Cre driven ablation of AR in myocytes caused significant reduction in body weight, LBM, and FBM in 16-week-old male mice. Although lean mass was reduced in myocyte-ARKO males, this was independent of changes to bone parameters as myocyte-ARKO did not affect trabecular or cortical bone outcomes measured by micro-CT. However, the authors did observe that myocytic AR seemed to regulate fiber-type and limb muscle masses variably, where myocyte-ARKO decreased mass of EDL, but not soleus, gastrocnemius, or quadriceps, and increased the presence of Type 1 fibers in soleus. Muscle function and oxidative capacity did not seem to be impaired by MCK-driven myocyte ARKO, as Ophoff and Van Proeyen et al. showed that even with a modest increase in slow-twitch fibers in soleus, neither succinate dehydrogenase (SDH) activity nor muscle tension and fatigability differed between myocyte-ARKO and control male soleus. In 2010, contrasting resulted were published by Chambon and colleagues [205] revealing that human alpha-skeletal actin (HSA)-Cre driven myocyte-ARKO did not affect TBM, or hindlimb mass of EDL, soleus, gastrocnemius, or tibialis anterior (TA) in male mice aged 6-, 13-, or 40-weeks. Yet functionally, myocyte-ARKO did result in a modest decline in grip strength beyond 10-weeks of age, and a reduced maximal isometric tetanic force in TA and EDL, but not soleus, in 20-week-old male mice. Similarly, Ghaibour et al. [208] supported these findings to show that myocytic AR had limited effect on lean or fat mass between 5 and 30 weeks of age in male mice yet was involved in the transcription of genes related to polyamine biosynthesis and oxidative metabolism. Other work had also shown that polyamine biosynthesis was at least in part mediated by myoblast- and myofiber-specific AR in α-actin- and MCK-Cre ARKO male mice at 12-weeks of age [204], and in HSA-Cre ARKO female mice at 13-weeks of age [209]. The dynamic nature of amino acid metabolism led to further questions regarding the role of muscle-AR in supporting muscle adaptation upon interaction with exercise or functional overload. However, neither myoblast- nor myofiber-specific ARKO impacted voluntary running wheel activity in 12-week-old male mice, even with the modest reductions in absolute and relative hindlimb muscle mass and elevations in WAT depot mass in those muscle-specific ARKO lines [183]. Additionally, HSA-mediated ARKO in myocytes did not affect overload-induced hypertrophy, Type 2 fiber transition, maximal force production, or fatiguability of plantaris following synergist ablation of soleus and gastrocnemius in 12-16-week-old male mice [206]. To date, muscle-specific ARKO studies had consistently used male subjects, limiting the conclusions surrounding sex differences in muscle-AR regulation of body. However, a recent study from Sakakibara and colleagues [209] using a female-only design, showed that myofiber-ARKO in females (similar to the muscle-ARKO body composition outcomes in males) did not change TBM or hindlimb muscle mass of TA, quadricep, or gastrocnemius at 13-weeks of age. Overall, skeletal muscle mass and function, as well as running activity and response to synergist ablation, are observed to be relatively stable in the absence of AR expression in the myocyte, myoblast, or myofiber. However, skeletal muscle-specific AR seems to maximize lean mass, muscle type-specific mass and fiber-type count, muscle strength and contractility in MCK-Cre but not HSA-Cre transgenic KO lines.

SCs were first described in the 1960’s in investigations hoping to identify the mechanism by which skeletal muscle gave rise to myonuclei and their role in proliferation of regenerating myofibers [233–235]. Seminal contributions in the SC literature [236–242] revealed the potential of these myogenic progenitor cells in activating gene transcription and cell signaling pathways associated with muscle development in utero and during postnatal life. Subsequently, SC were thought to mediate skeletal muscle capacity for adaptation and regeneration following exercise or injury, at least in part through co-localized AR [86, 243]. While androgen treatment promoted SC fusion in mature LA myofiber [244], myogenic lineage in pluripotent cells [245], and myoblast cell-cycling in an AR-dependent manner [246], the reliance of AR within SCs on regulating body composition and skeletal muscle mass in vivo was less clear. Using a MyoD-iCre driven ARKO in male mice, several works had outlined changes in post-pubertal lean and fat tissue in response to AR ablation in SCs [191, 192, 203]. Dubois and colleagues [191, 203, 247] first revealed that SC-ARKO did not affect TBM, hindlimb mass of TA, EDL, soleus, or gastrocnemius, as well as various subcutaneous and visceral fat depot mass in 12- and 20-week-old male mice. Similarly, Jardi et al. [192] found that hindlimb muscle mass was unchanged in response to SC-ARKO at 16-weeks of age, although modest declines were noted in BC/LA mass. In adulthood, loss of AR expression in SCs seemed to impact muscle strength from 16–52-weeks of age and voluntary activity at 16-weeks of age, but not twitch capacity or fatigue resistance in EDL and soleus [192, 203]. Although the phenotype of hindlimb muscle was largely unaffected by the loss of AR in SCs, it is clear that AR within SCs regulates BC/LA mass [192, 203], likely through gene expression of insulin-like growth factor 1 (*Igf-1*) and myostatin (*Mstn*). SC-ARKO resulted in significant reduction of Mstn expression [203, 247] with ChIP-Seq uncovering canonical AR binding sites at the *Mstn* locus [203]. Overall, AR expression in SCs may play a minor role in maintaining age-specific activity and muscle strength outcomes, with limited impacts on lean or fat body composition in males.

Most recently, ChIP-Seq was used to identify AREs on the *Igf-1* locus in skeletal muscle mesenchymal progenitors (ie., fibro-adipogenic progenitor cells, FAPs). Sakai and colleagues [248] ablated AR in mesenchymal progenitors by tamoxifen-induced excision driven by platelet-derived growth factor receptor α Cre-ER (PDGFRα-CreER) in young, adult, and aged male mice. While BC/LA mass was significantly reduced at the 12-week, 6-month, and 28-month timepoints in the PDGFRα-CreER-ARKO mice, hindlimb muscle mass (ie., TA and gastrocnemius) was only minorly reduced at 6 months. Neither hindlimb nor forelimb skeletal muscle morphology or function were significantly affected by the loss of AR in mesenchymal progenitor cells, as indicated by maintained grip strength, absolute mass, fiber diameter, myofiber count, SC count, or fiber type proportions between transgenic and wild-type (WT) mice at 12-weeks of age. Additionally, though mesenchymal progenitors have a role in adipogenesis, there was no effect of PDGFRα-CreER-ARKO on epididymal or subcutaneous WAT at 6 or 28 months of standard chow. AR expression is not solely limited to the myocyte within the array of cell populations in muscle fibers. Its observed expression in non-myocytic cell types, including but not limited to SCs, fibroblasts, endothelial cells, and mesenchymal progenitor cells supports a coordinated effort of androgenic action on skeletal muscle maintenance [85, 86, 207, 248].

However, the role of androgens in promoting mesenchymal progenitor cell commitment to myogenic lineage cannot be ruled out completely. Single cell RNA sequencing data reveals that skeletal muscle carries various non-myocytic cell types, including satellite cells, FAPs, immune T and B cells, and endothelial cells [249]. This heterogenous pool of different cell-types and their transcriptional and translational capacity may be influenced by androgen stimulation across the muscle fiber. Indeed, non-myocytic cell types, such as fibroblasts, satellite cells, CD34 + precursor cells, and vascular endothelial cells express AR [193]. Thus, it is possible that the coordination of androgen-mediated action in these cell types through AR-dependent or -independent action may contribute to overall changes in body composition. For example, Singh and colleagues [245] used pluripotent, mesenchymal C3H 10T1/2 cells, capable of differentiating into muscle, fat, cartilage, and bone, under graded testosterone and DHT treatment to observe the progression of cell differentiation. The group found that incubation of CH3 10T1/2 cells with testosterone or DHT dose-dependently increased the number and area of MyoD- and MHC-expressing myotubes and myogenic cells compared to vehicle-treated controls. Additionally, mesenchymal cells showed a testosterone- and DHT-mediated dose-dependent reduction in fat cell count and mRNA expression of adipogenic differentiation markers, PPAR-γ2 and C/EBP-α. Moreover, the commitment to myogenic lineage and inhibition of adipogenic lineage by androgenic treatment was reversed dose-dependently by bicalutamide incubation, highlighting that cell commitment here was regulated through AR.

Skeletal muscle adaptation is intrinsically linked to the nervous system through changes in patterns of muscle contraction (ie., daily physical activity, regimented exercise, immobility, injury, or disease). The involvement of the motor neuron and its innervated muscle fibers is indispensable in positive or negative stressor-induced changes to muscle mass, fiber hypertrophy, fiber-type transition, and strength. As AR is expressed in neuronal tissue, there is rationale in studying the role of androgen-AR action in mediating skeletal muscle and body composition adaptation, yet the number of studies on this tissue are limited [210, 211]. Using synapsin I-Cre-driven excision of AR in the central nervous system, Yu and colleagues [210] observed a late-onset increase in TBM from 28- to 32-weeks of age, as well as epididymal and retroperitoneal fat mass gain, adipocyte hypertrophy, and increased circulating leptin at 36-weeks of age in male neuron-ARKO mice compared to WT controls. Although this study did not measure the response of neuronal AR deficiency on skeletal muscle phenotypic or functional outcomes, the data supported a role in neuronal AR for mitigating an obesogenic phenotype in an age-dependent manner. Next, CaMKIIa-Cre-driven AR ablation in neurons of the cortex, forebrain, hypothalamus, and olfactory bulb reduced the mass but not force or fatiguability of gastrocnemius and EDL, but not soleus, at 6- and 12-weeks of age in males [211]. Here, Davey and colleagues found no differences between neuronal ARKO and WT control TBM or subcutaneous, renal, or gonadal fat pad mass at 6- or 12-weeks of age, even with a significant increase in circulating testosterone and LH. Yet neuronal AR seemed to heavily maintain voluntary activity levels at 12-weeks of age, which the authors conclude could be a result of the decreased gastrocnemius mass at 6-weeks or a possible AR-mediated effect on running motivation in these brain regions. Overall, the specific role of neuronal AR on body composition and skeletal muscle morphology remains unknown, but these works demonstrate that AR expression in specific brain and nervous tissue regions could regulate adiposity and fiber-type specific mass potentially through changes to physical activity motivation.

### Transgenic mutants: muscle-specific androgen receptor overexpression

AR gene ablation at global or cell-specific levels provided a fundamental understanding of how absent ligand-receptor activity, beginning at embryonic days, impacted body composition and skeletal muscle phenotype in young to middle adulthood. However, the generation of novel transgenic mice with excessive CAG repeats in the polyglutamine tract of AR to model Kennedy’s Disease highlighted the involvement of mutated AR in androgen-dependent muscle and motoneuron pathology [34, 250]. The interest in resulting phenotypic disturbances of overactive AR led to the generation of a transgenic mouse model with AR overexpression solely in skeletal muscle using a HSA promoter (HSAAR) [251]. In 2007, we showed that HSAAR male, but not female, viability at birth was associated with prenatal flutamide exposure, highlighting that overexpression of AR in mouse skeletal muscle caused androgen-dependent early death. Furthermore, surviving HSAAR males in two founding lines (L78 and L141, corresponding to transgene copy number, and AR mRNA and protein expression) revealed a relationship between greater AR expression and severity of disturbance in body mass, motor function, EDL muscle size, and EDL myofiber number at 10 to 75 weeks of age. Sex differences in HSAAR phenotypic regression was driven by differences in androgen circulation, as 9-days of testosterone treatment in L141 HSAAR female mice induced drastic declines in body mass and motor function, but not EDL atrophy [251]. In 2011, we later identified that AR overexpression in skeletal muscle of mice implicated oxidative metabolism in atrophied glycolytic muscle [252]. In 5- to 28-week-old HSAAR male mice, Johansen and colleagues [252] observed a reduction in EDL myofiber number and CSA, as well as increased presence of atrophied fibers and SDH staining, indicating greater mitochondrial presence. This work also detailed the first generation of a mouse *Tfm*/HSAAR transgenic cross, which produced viable offspring with non-functional AR in all tissues except for that of skeletal muscle, where functional AR overexpression remained. Using the *Tfm*/HSAAR mouse, Johansen et al. [252] revealed that 9-days of testosterone treatment at 17-weeks of age reduced TBM, open-field testing activity, grip strength, and stride length in only *Tfm*/HSAAR but not *Tfm* males, highlighting that muscle-specific AR was involved in body composition and motor function declines in the L141 males.

Skeletal muscle morphology and the involvement of mitochondria in the HSAAR mouse phenotype was further studied in Musa et al. [253] using electron microscopy and electron transport chain (ETC) activity assays in 13- to 37-week-old L78 and L141 male mice. The study observed that both HSAAR lines had reduced myofibril width and increased interfibrillar mitochondrial density in EDL, while HSAAR males from only L78 presented a fiber-type transition from fast-oxidative (FO) to fast-oxidative-glycolytic (FOG) and an increase in ETC complex activity in TA [253]. Sex differences in the progression of HSAAR-mediated muscle deficits were shown to be driven by differences in androgen circulation, as only testosterone-treated HSAAR females exhibited reduced myofibrillar width, and increased mitochondrial density, area, number, and activity of Complex I-IV in the ETC.

In 2009, Niel and colleagues [254] used a previously generated skeletal muscle-specific AR overexpression vector [114] to generate the transgenic HSAAR and *Tfm*/HSAAR cross in Sprague Dawley rats. The work revealed that AR in skeletal muscle plays a role in lean body composition, as DXA-measured LBM% was significantly greater in HSAAR and *Tfm*/HSAAR but not *Tfm* male rats, compared to WT littermates 6-week-old [254]. In contrast to the TBM deficits in HSAAR male mice, TBM was equivalent between WT and HSAAR male rats at 8- to 10-weeks of age [255] and 4- to 10-weeks of age [256]. Considering the role of SARMs, exogenous androgen treatment, and global ARKO in mediating changes to adiposity in both humans and rodents, Fernando and colleagues [256] studied male HSAAR, *Tfm*, and *Tfm*/HSAAR rats to unravel the specific effect of muscle-specific AR overexpression on male body composition and muscle and adipose morphology at pubertal age. The observed increase LBM% of HSAAR male rats was a result of significantly reduced absolute FBM, FBM%, WAT mass, and adipocyte CSA [256]. Although LBM and EDL fiber-type proportions were unchanged in HSAAR versus WT males, there was a modest hypertrophy of Type 2b fibers in EDL. Reductions in adiposity across HSAAR but not *Tfm* male rats were thought to be a result of increased oxidative metabolism, as HSAAR males exhibited increased activity of ETC Complexes I-IV in EDL, while activity was reduced in *Tfm* males [256]. Altogether, the HSAAR model in both mice and rats highlights the involvement of skeletal muscle AR in mediating body composition and mitochondrial metabolism in young adult males.

### Transgenic mutants: functional outcomes and responses to exercise and aging

Considering the importance of AR signaling in maximizing endurance and resistance training-mediated muscle hypertrophy [11] and the correlation between circulating androgens and sport performance [52, 257, 258], there is interest in understanding the role that AR plays in exercise adaptation, specifically in skeletal muscle. Data in global ARKO mice showed that functional AR signaling was important for maintenance of maximum force production in EDL but not soleus [178], and maintenance of sex differences in muscle-specific fatiguability and sprint time to exhaustion [178, 188] in young adult mice. Although others have shown limited effect of lost or reduced AR signaling on muscle tension or grip strength during single bout testing at 16-weeks of age [187, 192]. Tissue-specific loss of AR from nervous tissue [211], the myofiber [187, 205, 206, 209], SCs [203], or FAPs [248] yielded minor, if at all significant, reductions in muscle-specific and whole-body functional outcomes of force production and grip strength beyond 10-weeks of age in males and females [209]. To note, worsened grip strength did not always occur concomitantly to reduced force production or fatiguability of EDL or soleus [203]. A considerable limitation of using these types of measures to extrapolate the role of AR signaling in exercise response is their time course to effect skeletal muscle form and function.

Skeletal muscle remodeling (ie., change in myofiber CSA or muscle size) via exercise is a process which takes weeks to coordinate, and requires chronic and progressive stimulus to promote. Our group explored the effects of 9-weeks of chronic endurance wheel running on body composition and myofiber size of HSAAR male and female rats, showing that a tenfold or fivefold increase in male and female TA AR expression, respectively, was insufficient to change sex-specific and sex-independent exercise adaptations in lean and fat body composition [259]. As we showed previously [256], muscle-specific AR overexpression was sufficient to increase both male and female LBM and selectively increase glycolytic myofiber size in EDL compared to WTs [259]. Additionally, we showed that greater AR content of TA was moderately correlated to greater glycolytic myofiber size of trained males and females, which has been reported in resistance trained young men [10]. We extended these observations to understand how modulation of AR in skeletal muscle would impact development, sexual maturation, and growth of skeletal muscle across sexes through the rat lifespan. In this work, we showed that sex differences in lean mass, fat mass, and muscle mass (ie., soleus, TA, and EDL), which appear at pubertal age (ie., PND42), seem to be promoted and maintained with limited change in expression of endogenous skeletal muscle AR throughout the sexual development period [89]. Moreover, we show that muscle fiber-type specific reductions in endogenous AR expression during adulthood do not seem to dampen absolute skeletal muscle mass growth, leading us to speculate that large changes in skeletal muscle AR expression do not contribute to the growth and age-related changes to skeletal muscle mass. However, with HSAAR transgenic expression, 6- to 12-month-old males but not females show a 14% increase in TA and EDL mass—a response similar to that seen during 8–16 weeks of resistance training [260]. Overall, our work in the HSAAR rat highlights that supraphysiological expression of AR in skeletal muscle is alone sufficient to increase lean mass, and glycolytic-specific fiber size and mass in both sexes in an age-dependent manner, yet surprisingly does not seem to interact with chronic endurance training on body composition or muscle phenotype outcomes in young adulthood. Moreover, the dynamic expression of AR in skeletal muscle through the lifespan [88, 89] suggests that more work needs to be done to identify how various modalities of exercise (dynamic, chronic stimuli which elicit varied phenotypic and metabolic outcomes in skeletal muscle and other tissues depending on age and sex [261]) interact with AR signaling to coordinate functional and phenotypic changes to skeletal muscle and adipose.

### Female subjects in pharmacological and transgenic AR studies

The recognition of sex differences in sport performance is prehistoric, and far precedes the identification and synthesis of testosterone, the isolation of AR, and the publication of seminal works which detail the dose-dependent effects of androgens or SARMs on skeletal muscle remodelling. Modern sex categorization in sanctioned sporting events began in the 1966 European Track and Field Championships, and since then had taken on many shapes as mandated by the International Olympic Committee and International Amateur Athletics Federation (IAAF). While regulations allowing sex chromatin karyotyping in 1968, PCR-screening for the SRY gene, and validation of external genitalia were abolished in 1992 by the IAAF [262], sport governing bodies still relied heavily on athlete division by sex, with the factor of “fairness” and “ethical sport” focused on androgenic profiles [258]. Guidelines for serum testosterone circulation in female athletes were established in 2011, qualified at < 10 nmol/L, and lowered in 2018 to < 5 nmol/L [263]. Prior to these mandates, sex differences in performance outcomes of endurance sports and strength were found to be associated with male-specific pubertal timing. In 1940, Espenschade [264] analyzed the sprint, jump, brace test, throw distance, and broad jump performances from 11- to 17-year-old males and females, revealing that between these ages, female performance was unchanged, while male performance progressively increased. The bifurcation of athletic performance beyond 13-years of age in males and females was shown in several other primary works [265–267]. Handelsman [257] analyzed sport performance differences between the sexes and found that sex differences increased across several endurance events beyond 12-years of age, overlapping with male-specific elevation in circulating testosterone [268]. Beyond pubertal age, sex differences in sport performance persist [269]. The knowledge gained by studies of organizational and activational effects of androgens on tissue growth and performance address important questions regarding the fairness of transwomen inclusion in sport, with most works citing that male physiology and advantage in musculoskeletal and cardiovascular systems cannot simply be inhibited or reversed by gender-affirming estrogen therapy [270]. Jarin and colleagues [271] show that although testosterone levels of affirmed female adolescents were reduced from 391.7 ng/dL at baseline to 199.3 ng/dL beyond 6-months of therapy, these levels were still significantly higher than the 29.5 ng/dL of total testosterone in biological females at baseline. Considering this, and the prevalence of hyperandrogenism in female elite athletes [272], there is strong evidence that androgenic involvement in promoting and maintaining total body, bone, and skeletal muscle mass and strength would provide advantage in overall sport performance in transwomen versus biological female athlete peers.

A serious limitation in the AR transgenic literature is the lack of equal-sex representation within subjects, making it difficult to completely understand the role of functional AR in regulating female body composition. Beyond the fundamental knowledge gleaned from sex as a biological factor in basic physiology research, there is strong clinical relevance to study female response to exogenous androgens, SARMs, and transgenic AR manipulation. There seems to be greater prevalence of androgen dysregulation in elite-level female athletes via polycystic ovarian syndrome (PCOS) [273–276], and in experimental settings, women show responsiveness to androgen supplementation in an age- and dose-dependent manner [277–280], supporting the hypothesis that androgens and AR play some role in modulating female body composition and muscle function. Female skeletal muscle shows responsiveness to androgens throughout life, including prenatal and neonatal ages, where testosterone exposure results in masculinization and hypertrophy of androgen-dependent and -independent muscle [281–285].

The body of preclinical studies utilizing SARMs in female rodents has focused more on the capacity of AR to prevent or reverse menopause-induced bone loss and weakness [286–290], in comparison to the skeletal muscle-sparing outcomes in male SARM testing. Through AR, androgens play significant roles in female bone metabolism with flutamide-treated intact female rats displaying decreased femur mass, calcium content, and bone resorption rate [291]. The ovariectomy (OVX) model is used to induce a menopause-like bone phenotype, specifically osteopenia and accelerated bone loss in female rodents. However, this model in androgen- and AR-focused studies is not without its limitations, including a criticism of the gonadectomy approach where abrupt loss of circulating hormones via surgical intervention does not seem to mirror tissue phenotype and metabolism as observed in the gradual onset of menopause, hypogonadism, or skeletal muscle wasting diseases. OVX limitations extend beyond this with the loss of gonadal androgen production from excised ovaries, and residual ER and PR activity within the intact uterus and uterine horns with peripherally produced estrogens via brain or adipose. Additionally, in preclinical SARM work, the use of DHT for experimental controls could negatively regulate LH, FSH, and estrogen production in intact females. These factors add complexity to OVX-focused preclinical studies of exogenous androgens, SARMs, or ARKO in females, as it is difficult to isolate tissue phenotype deficits to a mechanism of action from either lost testosterone-AR, E2-ER, or progesterone-PR activity.

In the collection of global ARKO mouse studies with body composition outcomes (ie., TBM; skeletal muscle mass, fiber-type, or CSA; adipose mass or adipocyte CSA; or bone parameters), there are few works which utilize both male and female subjects, making it difficult to establish clear conclusions regarding sex differences in AR regulation of body composition. However, several works have detailed the effects of ARKO driven by CMV-Cre [175, 178, 179], ACTB-Cre [195], and PGK-Cre [292] on female body composition. Kawano and colleagues [175] were the first to show that CMV-Cre-driven ARKO reduces the body weight growth curve of males, but not females compared to WT littermates from PND24 to PND52. Using DXA and CT scanning on excised femur and tibia bones of 8-week-old male and female mice, Kawano et al. revealed significant bone loss of the femur in male but not female ARKO mice, as well as ARKO-induced increase in bone turnover in males only. Considering that aromatizable testosterone, but not DHT, replacement in GDX male ARKO mice improved femur BMD, it suggests that males too rely on estrogen-ER signaling for bone maintenance, yet not as critically as females, who experience little perturbance in bone morphology as a response to lost AR. However, Kang et al. [195] observed sex-equivalent reductions in skull bone volume and surface area in ACTB-Cre-driven ARKO male and female mice at 8-weeks. Sex differences were observed in femurs collected from 9-week-old CMV-Cre-driven ARKO mice, wherein male ARKO mice showed reduced TBM, trabecular bone volume, cortical bone thickness, and mineralizing surface %, while these outcomes did not differ between ARKO and WT females [179]. MacLean and colleagues [179] also revealed sex-equivalent responses to ARKO in bone as measured by micro-CT, including the reduction of femur trabecular thickness and periosteal/medullary circumference. Earlier, the same group identified a major sex-specific effect of ARKO on hindlimb skeletal muscle mass such that 9-week-old male, but not female, ARKO mice exhibited a 12.6% reduction in total mass and a 22–25% reduction in TA, EDL, soleus, and gastrocnemius absolute mass [178]. The limited effects of ARKO on significant disturbance of female body composition were further highlighted by Fagman et al. [292] who showed that female PGK-Cre-driven ARKO mice did not differ in body weight growth from 4- to 16-weeks of age, or in DXA-measured lean or fat body mass at 15-weeks of age. Furthermore, excised visceral mesenteric fat and subcutaneous inguinal fat depot masses were not affected by ARKO in 15-week-old female mice. Overall, although functional AR in females may be needed for some bone maintenance in 8- to 9-week-old mice [179, 195], it seems that female total mass [175, 179], hindlimb skeletal muscle, and adipose mass rely less on global AR presence [178, 292]. However, more work is needed to establish the role of AR in maintaining female phenotypic outcomes at varying ages.

### Limitations and future directions

The collective AR transgenic literature which focuses on the steroid receptor’s role in modulating body composition and tissue phenotype has consistently measured outcomes of AR manipulation in hindlimb skeletal muscles. However, considering the differences in embryonic origin of skeletal muscle groups, and their different phenotypic, metabolic, and activation profiles, there are likely differences in the level of their reliance on AR in sex-specific tissue development and maintenance. Our supraphysiological muscle-specific AR work using the HSAAR model and the hypophysiological work of others [183, 187, 205, 209] had focused on CSA, mass, and metabolic changes within EDL, TA, and soleus due to several methodological advantages, including simplicity of dissection, relatively homogenous fiber-types, and standardization in the muscle/exercise physiology fields. However, this approach has, and continues to, severely limit the understanding of AR action within other skeletal muscles (ie., axial/postural muscles, upper limb muscles, and voluntary portions of diaphragmatic muscle). Further examination of the development and maintenance of sex differences in mass, myofiber size, oxidative/glycolytic capacity in these muscles is required to understand how AR across a wider variety of muscle groups is involved in growth and chronic exercise adaptation.

Although it is tempting to extrapolate the results of HSAAR expression on gains in adulthood muscle mass and reduction of adiposity to the body composition outcomes facilitated by SARMs, there are several reasons why this may not be productive. Much of the pre-clinical animal data across SARM studies was done in GDX males under the rationale of age- or disease-related decline of total testosterone, however, aging in healthy individuals imparts seemingly modest changes to circulating testosterone in a chronic fashion [51]. Thus, the physiologically relevant growth and aging methods used in this literature (ie., gonadally intact animals) would result in different lean and fat tissue adaptations considering SARMs in GDX models completely replace lost androgens, while HSAAR-mediated AR protein increases likely supplement existing androgens in circulation. Furthermore, nonsteroidal SARMs were modelled after anti-androgen drugs, and as such, they have ligand-like activity on existing cytoplasmic or membrane-bound AR, much like steroidal SARMs. Whether SARMs modulate translation of AR protein at the level of skeletal muscle and adipose to increase respective anabolic and catabolic capacity is unclear. One case report of a young resistance-trained male self-administering two different SARM compounds for 5-weeks described decreased skeletal muscle AR content and increased intramuscular DHT and testosterone concentration when compared to trained, non-user males [293]. Moreover, SARM use, unlike HSAAR expression, showed a trend to decrease endogenous total and free testosterone levels in men [294]—a somewhat expected consequence of negative HPG feedback.

Gene ablation and overexpression likely impart a multitude of consequences across the genome. Although the altered phenotypic and cell signaling response in tissues following genetic knockout hopes to conclude a targeted mechanism of action for the binary removal of a single gene, this idealistic conceptualization of the mutant negates the thousands of genes altered following ablation [295, 296]. While genetic knockout substrains show genotypic and phenotypic pathways for mitigating loss of a target gene (ie., evolved similar phenotypes or secondary mutations in off-target genes), gene overexpression is perhaps more complicated in that the amount of target protein expression is highly variable, alongside the possible genome-wide effects of the mutation.

We and others have used gonadally intact rats to understand the physiological action of wild-type AR and transgenic AR on body composition, muscle, adipose, and bone outcomes. While orchidectomy (ORX), OVX, or anti-androgen treatment during sensitive growth periods or milestones in the lifespan would confirm a causal relationship between changes in circulating androgens, not tissue-specific AR, as the driving force of sex differences in the outcomes measured, there are considerable caveats to these methods as well. Pharmacological AR inhibitors do not have tissue specificity in vivo, thus phenotypic alterations might result from loss of AR action in any number of targets, rather than being specific to skeletal muscle or other tissue. Conversely, the effects of interacting cells, tissues and organ systems in vivo are unlikely to be recapitulated in 2D or 3D tissue models of skeletal muscle, even though flutamide + androgen treatment in those experiments would be targeted to muscle only. Notable among these issues in studies of sex differences are that cell line models limit the major impact of other androgenic and non-androgenic endocrine mechanisms. OVX, while removing androgens produced by the ovarian theca cells, also entails near total loss of circulating estradiol, which would have significant impact on adiposity and skeletal atrophy. Adrenalectomy in female rodents, although removing considerable production of DHEA, and thus peripheral conversion of testosterone, would not seem to be an improved methodology due to the subsequent loss of other peptide and steroid hormones produced within, including, epinephrine, norepinephrine, cortisone, cortisol, and aldosterone. Overall, the methodology for reducing or removing production of only circulating androgens in both sexes to compare androgen-mediated modulation of non-reproductive tissues in vivo is fairly limited.

## Conclusions

Normal development and growth of skeletal muscle, adipose depots, and the skeletal system are integral for healthy body composition and improved healthspan outcomes. Sex differences in these tissues arise at various stages of the lifespan, yet their regulation by androgens and AR is complex considering the ubiquity of AR expression. The importance of understanding how body composition is regulated in response to biological determinants (ie., gonadal steroid circulation and AR protein expression), environmental challenges (ie., physical activity and diet), and their interactions is clear in the clinical space where androgen administration to older men can improve LBM, especially when used as an adjunct therapy to resistance training. However, rodent studies have allowed identification of embryonic roles of global- and tissue-specific AR in regulating tissue phenotype at various ages across the lifespan. In post-pubertal male rodents, global- and cell-specific AR knockouts or overexpression showed causal effects on voluntary activity, acute endurance capacity, and muscle strength. Myocyte, myofiber, and SC AR seem to coordinate some outcomes of acute muscle function and skeletal muscle mass, with somewhat limited effects on TBM or adipose depots. Osteoblast AR seems to contribute to remodeling of trabecular bone but less so cortical bone, with limited effects on TBM. Finally, adipose AR may contribute to signaling pathways regulating lipid mobilization and utilization, although its effects on body composition seem to be limited at the measured timepoints of collection.

As a nuclear steroid hormone receptor, AR’s ligand-dependent action makes it a target for regulating some aspects of tissue anabolism and catabolism, thus contributing to the complex systems regulating tissue homeostasis of muscle, fat, and bone. Here, we summarized the currently understood effects of androgens, SARMs, and embryonic AR transgenic models on changes in body composition and muscle, adipose, and bone phenotype, and present areas for future work to help identify more distinct roles for AR in regulation of tissue morphology.

## Supplementary Information

Additional file 1.

## Data Availability

No datasets were generated or analysed during the current study.

## Abbreviations

E2: 17 β Estradiol
3β-HSD, 17β-HSD: 3β- And 17β-hydroxysteroid dehydrogenase
S6K1: 40S ribosomal protein S6 kinase 1
DHT: 5α-Dihydrotestosterone
ACTB: β-Actin
aP2: Adipocyte Protein 2
ATGL: Adipose triglyceride lipase
AIS: Androgen insensitivity syndrome
AR: Androgen receptor
ARKO: Androgen receptor knockout mouse
ARE: Androgen-response elements
ArKO: Aromatase knockout
BPH: Benign prostatic hyperplasia
BMC: Bone mineral content
BMD: Bone mineral density
BAT: Brown adipose tissue
Ca2 +: Calcium
CREB: CAMP response element-binding protein
CRPC: Castration-resistant prostate cancers
C/EBP-δ, C/EBP-α: CCAAT-enhancer-binding proteins
ChIP-Seq: Chromatin immunoprecipitation sequencing
CT: Computed tomography
Cre: Cre recombinase
CSA: Cross-sectional area
LBD: C-terminal ligand-binding domain
DHEA: Dehydroepiandrosterone
DAG: Diacylglycerol
DBD: DNA-binding domain
DXA: Dual-Energy X-ray Absorptiometry
ETC: Electron transport chain
ER: Estrogen receptor
ESR1: Estrogen receptor alpha
4EBP1: Eukaryotic initiation factor 4E-binding protein 1
EDL: Extensor digitorum longus
ERK1/2: Extracellular signal-regulated kinase 1/2
FO: Fast-oxidative
FOG: Fast-oxidative-glycolytic
FBM: Fat body mass
CPT1: Fatty acid mitochondrial transporter
FAS: Fatty acid synthase
FAPs: Fibro/adipogenic progenitors
FSH: Follicle-stimulating hormone
FOXK1: Forkhead box K1
FCG: Four Core Genotypes model
GR: Glucocorticoid receptor
GDX: Gonadectomy
GnRH: Gonadotropin-releasing hormone
Hsp: Heat shock protein
HSL: Hormone sensitive lipase
HAS: Human alpha-skeletal actin
CMV: Human cytomegalovirus
IP: Inositol phosphates
IP3: Inositol triphosphate
Igf-1: Insulin-like growth factor 1
IAAF: International Amateur Athletics Federation
KLF: Krüppel-like factors
LBM: Lean body mass
BC/LA: Levator ani/bulbocavernosus
LPL: Lipoprotein lipase
LH: Luteinizing hormone
MTORC1: Mechanistic target of rapamycin complex 1
MR: Mineralocorticoid receptor
MAPK: Mitogen activated protein kinase
MAG: Monoacylglycerol
MCK: Muscle creatine kinase
MPS: Muscle protein synthesis
HSAAR: Muscle-specific AR overexpression
MyoD: Myoblast determination protein 1
MEF2c: Myocyte enhancer factor 2c
MHC: Myosin heavy chain
Mstn: Myostatin
NTD: NH_2_-terminal transcriptional regulation domain
NLS: Nuclear localization signal
ORX: Orchidectomy
OVX: Ovariectomy
loxP: P1 phage
PPAR-γ: Peroxisome proliferator-activated receptor gamma
PGC1α: Peroxisome proliferator-activated receptor gamma coactivator 1-alpha
PA: Phosphatidic acid
PI3K: Phosphatidylinositol-3 kinase
PGK: Phosphoglycerate kinase 1
PDGFRα-CreER: Platelet-derived growth factor receptor α Cre-ER
PCOS: Polycystic ovarian syndrome
PND: Post-natal day
PR: Progesterone receptor
Akt: Protein kinase B
PKA/PKC: Protein kinases A/C
Src: Proto-oncogene tyrosine protein kinase
Col1a1: Rat type 1a1 collagen promoter
RSPO2: R-spondin 2
SC: Satellite cells
SARM: Selective androgen receptor modulators
SRF: Serum response factor
α-actin: Skeletal alpha actin
SBMA: Spinal and Bulbar Muscular Atrophy
SHBG: Steroid hormone binding globulin
SREBF: Sterol regulatory element binding factor
SDH: Succinate dehydrogenase
TA: Tibialis anterior
Tfm: Testicular feminization mutation
TBM: Total body mass
TEM: Transmission Electron Microscopy
UCP2: Uncoupling protein 2
WAT: White adipose tissue
WT: Wild-type
